# New Poly(lactic acid)–Hydrogel Core–Shell Scaffolds Highly Support MSCs’ Viability, Proliferation and Osteogenic Differentiation

**DOI:** 10.3390/polym15244631

**Published:** 2023-12-06

**Authors:** Chiara Pasini, Stefano Pandini, Federica Re, Matteo Ferroni, Elisa Borsani, Domenico Russo, Luciana Sartore

**Affiliations:** 1 Materials Science and Technology Laboratory, Department of Mechanical and Industrial Engineering, University of Brescia, via Branze 38, 25123 Brescia, Italy; c.pasini012@unibs.it (C.P.); stefano.pandini@unibs.it (S.P.); 2 Unit of Blood Diseases and Bone Marrow Transplantation, Department of Clinical and Experimental Sciences, University of Brescia, ASST Spedali Civili, Piazzale Spedali Civili 1, 25123 Brescia, Italy; federica.re@unibs.it (F.R.); domenico.russo@unibs.it (D.R.); 3 Department of Civil, Environmental, Architectural Engineering and Mathematics (DICATAM), University of Brescia, Via Valotti 9, 25123 Brescia, Italy; matteo.ferroni@unibs.it; 4 National Research Council (CNR)—Institute for Microelectronics and Microsystems, Bologna, Via Gobetti, 101, 40129 Bologna, Italy; 5 Division of Anatomy and Physiopathology, Department of Clinical and Experimental Sciences, University of Brescia, 25123 Brescia, Italy; elisa.borsani@unibs.it

**Keywords:** scaffold design, PLA, gelatin–chitosan hydrogel, tissue engineering, bone regeneration, 3D printing

## Abstract

Scaffolds for tissue engineering are expected to respond to a challenging combination of physical and mechanical requirements, guiding the research towards the development of novel hybrid materials. This study introduces innovative three-dimensional bioresorbable scaffolds, in which a stiff poly(lactic acid) lattice structure is meant to ensure temporary mechanical support, while a bioactive gelatin–chitosan hydrogel is incorporated to provide a better environment for cell adhesion and proliferation. The scaffolds present a core–shell structure, in which the lattice core is realized by additive manufacturing, while the shell is nested throughout the core by grafting and crosslinking a hydrogel forming solution. After subsequent freeze-drying, the hydrogel network forms a highly interconnected porous structure that completely envelops the poly(lactic acid) core. Thanks to this strategy, it is easy to tailor the scaffold properties for a specific target application by properly designing the lattice geometry and the core/shell ratio, which are found to significantly affect the scaffold mechanical performance and its bioresorption. Scaffolds with a higher core/shell ratio exhibit higher mechanical properties, whereas reducing the core/shell ratio results in higher values of bioactive hydrogel content. Hydrogel contents up to 25 wt% could be achieved while maintaining high compression stiffness (>200 MPa) and strength (>5 MPa), overall, within the range of values displayed by human bone tissue. In addition, mechanical properties remain stable after prolonged immersion in water at body temperature for several weeks. On the other hand, the hydrogel undergoes gradual and homogeneous degradation over time, but the core–shell integrity and structural stability are nevertheless maintained during at least 7-week hydrolytic degradation tests. In vitro experiments with human mesenchymal stromal cells reveal that the core–shell scaffolds are biocompatible, and their physical–mechanical properties and architecture are suitable to support cell growth and osteogenic differentiation, as demonstrated by hydroxyapatite formation. These results suggest that the bioresorbable core–shell scaffolds can be considered and further studied, in view of clinically relevant endpoints in bone regenerative medicine.

## 1. Introduction

Polymeric scaffolds have been studied with increasing interest for tissue engineering applications, thanks to the availability of a wide range of biocompatible and biodegradable polymers and to the development of several convenient processes for manufacturing interconnected porous structures, e.g., by means of porogenic agents, electrospinning or 3D printing [1,2]. These biomaterials act as three-dimensional scaffolds able to mimic the functionality of the native extracellular matrix (ECM) in the human body and to support cells and guide them towards a specific tissue construct [1]. Aiming to mimic the physicochemical properties and biological activity of native extracellular matrix, both synthetic and natural-based materials have been employed. Given the complex functionalities of the ECM, the most promising results have been achieved with composite scaffolds combining multiple material classes. In fact, the mechanical and degradation behavior of synthetic polymers is typically easier to control and tailor according to the specific target tissue; on the other hand, bioactivity can be greatly improved by using natural polymers, particularly in the form of hydrogel macromolecular networks [3,4,5].

Especially when dealing with mineralized tissues, it is important to provide adequate mechanical support until new tissue is formed [6]. Matching the mechanical properties of native tissue is also believed to stimulate proper cell differentiation [7]. For these reasons, several scaffolds have been realized with stiff thermoplastics, above all biocompatible and biodegradable polyesters, such as poly(lactic acid) (PLA) and poly(ε-caprolactone) (PCL) [8]. These materials have been combined with bioceramics (e.g., tricalcium phosphate, hydroxyapatite) and/or hydrogels (e.g., gelatin, chitosan, alginate, hyaluronic acid) to enhance the scaffold bioactivity through multiple solutions, including surface-modified polymers [9], embedded particles/nanoparticles [10,11], co-axial fibers [12,13], interpenetrating/semi-interpenetrating networks [14] and other composite systems [15,16,17]. Moreover, tissue regeneration can be promoted by adding cells and growth factors to the scaffolds [18].

Mesenchymal stromal cells (MSCs) are the most frequently used stem cells in regenerative medicine approaches, thanks to their immunomodulation, tissue regeneration, and protective functions [19]. MSCs can be derived from different sources, such as bone marrow, adipose tissue, and oral tissues, and they can differentiate into various mesodermal cells, such as bone and cartilage lineages. It is noteworthy that the paracrine activity of MSCs is one of the most important features that contribute to tissue regeneration [20]. To consider these cells in different clinical situations, MSCs must fulfill the definition criteria requested by the International Society for Cellular Therapy (ISCT) [21].

In a previous work [22], the authors developed a solvent-free manufacturing process for the realization of composite scaffolds for bone regeneration, grafting a stiff PLA–PCL porous core [23] with a bioactive gelatin–chitosan hydrogel shell [24], which was found to form a thin layer even into the innermost pores. While the PLA-based core allowed obtaining stiffness and strength comparable with those of mineralized tissues, the shell was grafted on its surface with the aim of promoting cell spreading, proliferation and osteogenic differentiation. Noteworthily, in vitro experiments with human bone marrow mesenchymal stromal cells (BM-hMSCs) proved that the composite scaffolds could also host viable cells and support cell proliferation and osteogenic differentiation.

Based on the recent rise of additive manufacturing (AM) in tissue engineering, the authors deemed it interesting to improve the possibility to tailor their core–shell scaffolds by producing the core through fused deposition modeling (FDM). Indeed, AM technologies allow accurate reproduction of anatomical shapes as well as easy customization of the scaffold internal structure [25,26,27], but few geometrical configurations have been studied so far (0°/90° lay-up [28,29], simple cubic cells [30,31], and a few alternative designs [32,33]). In particular, the design optimization of core–shell scaffolds incorporating a hydrogel within a stiff polymer core would require deeper knowledge of their structure–property correlations [34,35,36]. Therefore, to study the influence of the core structural arrangement on scaffold properties, the authors produced 3D-printed PLA cores with several lattice structures and integrated them with hydrogel [37]. The influence of lattice geometrical parameters on the hydrogel content, stiffness and strength of the scaffolds was investigated, also considering anisotropic effects due to external shape, lattice strut arrangement and printing direction. The tunability of the scaffold properties was highlighted and the results appeared especially promising in view of bone tissue engineering.

In this work, composite scaffolds with a PLA core and a gelatin–chitosan hydrogel shell were prepared by FDM of PLA lattice structures and subsequent grafting of the bioactive hydrogel, presenting highly interconnected pores obtained by freeze-drying. Such hybrid structures were developed aiming at mimicking the hierarchical structure of bone tissue. Three different types of lattices, hosting different amounts of hydrogel, were investigated. Special attention was dedicated to the study of mechanical properties provided by the core and of the effect of hydrolytic degradation on the scaffold strength, stiffness, and hydrogel stability. Finally, the composite materials were seeded with hBM-MSCs and subjected to cell viability, proliferation, morphology and mineralization assessments. Thanks to the core–shell design, these scaffolds may ensure long-term temporary mechanical support, owing to their stiff core, as well as a favorable environment for cell growth and colonization throughout the hydrogel, which is destined to be replaced faster by new tissue during the regeneration process. Moreover, the overall performance and resorbability of the scaffolds can be easily modulated by properly tailoring their 3D-printed core structure and the associated core–shell proportions.

## 2. Materials and Methods

### 2.1. Materials

Poly(L-lactic acid) (Raise3D PLA Premium filament, density 1.2 g/cm) was kindly supplied by Raise 3D Technologies, Inc., (Irvine, CA, USA).

Pharmaceutical grade type A gelatin (280 bloom, viscosity 4.30 mPs) was provided by Italgelatine (Cuneo, Italy). Poly(ethylene glycol) diglycidyl ether (PEGDGE) (molecular weight 526 Da) and chitosan (molecular weight 50,000 ÷ 190,000 Da, degree of deacetylation 75–85%) were supplied by Sigma-Aldrich Co., (Milan, Italy). Ethylene diamine (EDA) and acetic acid were provided by Fluka (Milan, Italy).

Dulbecco’s modified Eagle’s medium (DMEM) and fetal bovine serum (FBS) were purchased from Sigma-Aldrich, Milan, Italy.

### 2.2. Preparation of Core–Shell Composite Scaffolds

**Figure 1:**
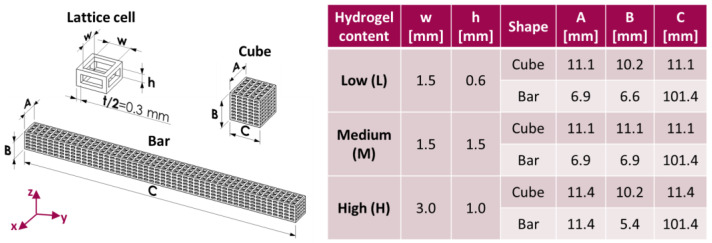
Hole dimensions and global dimensions for lattice-structured cubes and bars capable of hosting low, medium or high hydrogel content values.

Three types of lattice core structures were designed by means of the software Solidworks (SOLIDWORKS® Education Edition 2020, Dassault Systèmes, Vélizy-Villacoublay, France), adopting the cartesian coordinate system in Figure 1. All structures had the same strut thickness (t = 0.6 mm) but different hole width (w) along x and y and different hole height (h) along z, properly chosen in order to control the amount of hydrogel that may be hosted by the lattice (L: low; M: medium; H: high). Cubic specimens were employed in physical–mechanical characterizations and degradation experiments, while bar specimens were produced for biological characterizations. The outer dimensions of the specimens (A, B, C), as well as the lattice parameters (w, h, t), are all summarized in Figure 1 for the different types of structures realized.

The core specimens were realized by FDM, using the PLA filament, slicing software (IdeaMaker version 4.3.2) and a 3D printer (Raise3D Pro2), all provided by Raise 3D Technologies. The slicing of the CAD models was performed adopting z as build direction, with a layer thickness of 0.1 mm and without adding any supports. A 0.2 mm nozzle was employed, and the nozzle temperature and the bed temperature were set to 205 °C and 60 °C, respectively.

For the hydrogel shell preparation, 6 g of gelatin were dissolved in 65 mL of distilled water at 40 °C under mild magnetic stirring, followed by 1.4 g of PEDGE, 32.5 g of a chitosan solution (2 wt% in acetic acid 1%), and 70 mg of EDA. The reactants were left dissolving and reacting at 40 °C under stirring for about 10 min, during which grafting/crosslinking began through condensation reactions between the epoxy groups of PEGDGE and the amino groups of gelatin and chitosan. According to the amounts of ingredients employed, the nominal composition of the dry hydrogel was 74.3 wt% gelatin, 17.6 wt% PEGDGE and 8.1 wt% chitosan.

The lattice core structures were immersed in the hydrogel forming solution at 40 °C, and penetration of the hydrogel into their holes was ensured by applying three cycles of vacuum/air, which helped prevent entrapped air bubbles. After about 40 min at 40 °C followed by 20 min at room temperature, the scaffolds were subjected to freezing and freeze-drying in a lyophilizer (HyperCOOL HC3055, LabTech Srl, Sorisole, Italy). Dry core–shell specimens were freed from the excess hydrogel and crosslinking reactions were completed by post-curing in an oven at 45 °C under vacuum for 2 h. Unreacted reagents were finally removed by washing with distilled water, a further freeze-drying treatment was carried out to prevent hydrolysis during storage, and the final hydrogel content was evaluated by weight.

### 2.3. Physical and Mechanical Characterization

The scaffolds were observed under an optical microscope (Leica DMS 300, Leica Microsystems, Wetzlar, Germany), both in dry conditions and after immersion in distilled water for 24 h.

The theoretical void volume (V_v,th_) of the core lattices was obtained from CAD models, while their experimental void volume (V_v,exp_) was evaluated as follows:

(1) $$V_{v,exp}\left[\%\right]=\left(1-\frac{V_{c}}{V}\right)\times 100$$

where V_c_ is the core volume and V is the total volume (i.e., A × B × C, according to Figure 1). V_c_ corresponds to m_c_/ρ_c_, where m_c_ is the core mass (measured by laboratory balance: Gibertini E42-B, Gibertini Elettronica, Novate Milanese, Italy) and ρ_c_ is the density of Raise3D PLA Premium.

Theoretical and experimental values of hydrogel content (H_th_, i.e., the maximum amount that may be incorporated; H_exp_, i.e., the amount actually incorporated) in the scaffolds were calculated by weight through the following equations:

(2) $$H_{th}\left[\%\right]=\frac{{\rho_{s}V}_{v,exp}}{\rho_{c}\left(100-V_{v,exp}\right)+\rho_{s}V_{v,exp}}\times 100$$

(3) $$H_{exp}\left[\%\right]=\frac{m_{cs,dry}-m_{c}}{m_{cs,dry}}\times 100$$

where ρ_s_ is the density of the hydrogel and m_cs,dry_ is the mass of the core–shell specimens in dry conditions.

The water uptake (W) of the core–shell specimens was obtained as:

(4) $$W\left[\%\right]=\frac{m_{cs,wet}-m_{cs,dry}}{m_{cs,dry}}\times 100$$

where m_cs,wet_ is the mass of the core–shell specimens after immersion in water for 24 h.

Compression tests were carried out at room temperature on cubic core and core–shell specimens by means of an electromechanical dynamometer (Instron 3366, Illinois Tool Works Inc., Norwood, MA, USA), equipped with a 10 kN load cell. The load was applied along the transverse direction of the 3D-printed lattices (i.e., x- or y-direction in Figure 1), setting the crosshead speed to 2 mm/min. From load (P) and displacement (u) data, apparent stress (σ_app_) and apparent strain (ε_app_) were calculated through the following equations:

(5) $$\sigma_{app}\left[MPa\right]=\frac{P}{S}$$

(6) $$\varepsilon_{app}\left[mm/mm\right]=\frac{u}{A}$$

where S is the total area of the specimen cross-section (i.e., A x × x B, according to Figure 1) and A is the cubic specimen height along the compression direction. The term apparent indicates that stress and strain are here evaluated as macromechanical parameters for a homogeneous equivalent material, while local stress and strain are not uniform due to the non-homogeneous structure of the specimens. Finally, compressive stiffness and strength were expressed in terms of apparent modulus (E_app_, corresponding to the initial slope of σ_app_ vs. ε_app_ curves) and apparent stress at failure (σ_app,f_, evaluated at the first peak or knee of the curves), respectively.

### 2.4. Hydrolytic Degradation Experiments

The degradation of core–shell scaffolds was studied in distilled water at 37 °C for a total of 7 weeks. The distilled water medium was changed every week.

The evolution of mechanical properties with degradation time was investigated by compression tests on specimens withdrawn at different time points (1 day, 21 days, 35 days, 49 days). Compressive stiffness and strength along degradation time were obtained as described in the previous paragraph.

The mass loss of the specimens was also recorded on scaffolds picked up from the bath at different time points (1 day, 7 days, 21 days, 35 days, 49 days) and dried under vacuum in an oven at 40 °C. It was expressed both as percentage of the initial mass of the dry core–shell specimen and as percentage of the initial mass of the dry hydrogel shell:

(7) $$mass\;loss/total\;mass\left[\%\right]=\frac{m_{cs,dry}-m_{deg}}{m_{cs,dry}}\times 100$$

(8) $$mass\;loss/hydrogel\;mass\left[\%\right]=\frac{m_{cs,dry}-m_{deg}}{m_{cs,dry}-m_{c}}\times 100$$

where m_deg_ is the mass of the specimens after degradation and drying.

### 2.5. In Vitro Characterization, Optical and Electron Microscopy

The bar-shaped core–shell specimens were cut along their length into about 20 slices each, obtaining scaffolds with a thickness of about 5 mm. The scaffolds were packed under vacuum into polypropylene bags and sterilized by gamma irradiation with a dose of 25 kGy of Cobalt 60 gamma rays, according to UNI EN ISO 11137 standard [38] for the sterilization of health care products.

#### 2.5.1. BM-hMSC Culture, Seeding and Osteogenic Differentiation

For the purpose of the study, commercial BM-hMSCs (PromoCell, Heidelberg, Germany) were expanded, as previously described [39]. All experiments were conducted with cells between passage 3 and 4.

Dry scaffolds were placed in a 24-well non-adherent plate (Corning, Sigma-Aldrich, St. Louis, MO, USA) for cell seeding. The cell concentration was adapted to have the required number of cells in a volume of 50μL of growth medium (GM), consisting of DMEM medium supplemented with FBS. A small drop (7 × 10^5^/50 μL viable BM-hMSCs) was slowly deposited on the top of each dry scaffold, waiting for 2 h at 37 °C for the complete absorption of the drop. After this time, 1 mL of GM was added to each well. The cell culture medium was changed three times per week. Each construct was analyzed on day 28 for cell viability and cell proliferation.

Additionally, we seeded BM-hMSCs in the PLA-CH(L), PLA-CH(M) and PLA-CH(H) to investigate the osteogenic differentiation capability of the scaffolds at day 28, following the protocol previously published [39]. Particularly, BM-hMSCs were grown in osteogenic medium (OM) for 28 days.

#### 2.5.2. BM-hMSC Viability and Proliferation Assay

A live/dead kit for mammalian cells (ThermoFisher, Waltham, MA, USA) was used to evaluate the cell viability of the BM-hMSCs in the scaffolds. The samples were washed with DPBS and incubated for 30–45 min at room temperature in DPBS with 2 μM of calcein AM and 4 μM of ethidium homodimer-1 (EthD-1). NucBlue^®^ Live reagent (2 drops/mL) for nuclei staining was added to the cultures. An analysis of live (stained in green with calcein AM) and dead (stained in red with EthD-1) cells was then performed using a Zeiss Observer Z1 fluorescence microscope (Jena, Germany).

Cell proliferation was determined using the Cell Counting Kit-8 (CCK-8, Sigma-Aldrich, USA) on day 28 of cell culture, following the manufacturer’s instructions. Briefly, the cell-cultured samples (three replicates) were moved to a new cell culture plate and incubated with a fresh culture medium containing the CCK-8 reagent (ratio 1:10) at 37 °C for 2 h 30 min. Then, the absorbance of 100 μL of supernatant transferred to a new cell culture plate was measured at 450 nm with an Infinite 200 PRO plate reader (Tecan, Switzerland). Absorbance at 450 nm is proportional to the number of viable cells in each sample.

#### 2.5.3. Histomorphological Analysis at Optical Microscope

Scaffolds were embedded in paraffin using an automatic processor Donatello series 2 (Diapath S.p.A., Bergamo, Italy). Serial paraffin sections (5 µm thick) of each sample were cut with a semiautomatic microtome Galileo semi-series 2 (Diapath S.p.A., Bergamo, Italy). Alternate sections were deparaffinized and rehydrated, according to standard procedures and stained with hematoxylin–eosin stain (automatic stainer Giotto; Diapath S.p.A., Bergamo, Italy) for general morphology. The micrographs were recorded using a camera equipped with an image analysis system (Image-Pro Premier 9.1; 2018, Immagini e Computer, Milan, Italy).

#### 2.5.4. Scanning Electron Microscopy Observation

Scanning electron microscopy (SEM) was used to evaluate both the adhesion and the osteogenic differentiation of the cells. For SEM observation, samples have been progressively dehydrated through immersion in alcohol solutions, with no additional preparation and without deposition of a conductive metal coating. The SEM (EVO LS-10 manufactured by ZEISS) was operated in environmental mode, i.e., at 0.1–0.2 Torr pressure at the specimen. The electron beam was accelerated at 15 keV, and the imaging detector was sensitive to electrons backscattered by the specimen.

### 2.6. Statistical Analysis

Statistical analysis for cell proliferation assay data was performed using one-way ANOVA with Tukey’s multiple comparisons test. Numerical results are presented as mean ± standard deviation (SD). Graphical results were produced using GraphPad Prism (version 7). Three replicates of each sample were used. Statistical significance was accepted at the probability level *p* < 0.05.

## 3. Results and Discussion

### 3.1. Physical and Mechanical Characterization

**Scheme 1:**
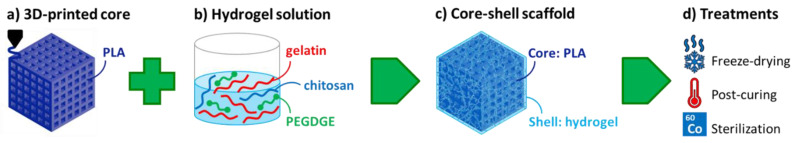
Fabrication process of composite core–shell scaffolds.

The fabrication process of core–shell composite scaffolds is displayed in Scheme 1. The core is a PLA lattice structure produced by additive manufacturing (a), providing the possibility to tailor both its mechanical properties and the hydrogel content, by varying the lattice strut arrangement and void volume fraction. The shell is created by synthesis of a hydrogel forming solution (b), which is grafted onto the core to obtain the composite scaffold (c). Final treatments include a freeze-drying step to create interconnected pores in the shell through ice sublimation, a post-curing treatment to complete crosslinking reactions, and, for scaffolds destined to in vitro experiments, sterilization by ^60^Co gamma rays. At the end of this process, the scaffold exhibits a hierarchical nested structure, where the PLA core constitutes the larger mesh, encompassing the smaller hydrogel mesh.

Core lattices are designed with the same strut thickness but different hole sizes, so that they may host different amounts of hydrogel. The width (w) and height (h) of the holes are defined in Figure 1, together with the overall dimensions of the specimens. Composite scaffolds with low, medium and high hydrogel content are from here on indicated as PLA-CH(L), PLA-CH(M) and PLA-CH(H), respectively.

**Figure 2:**
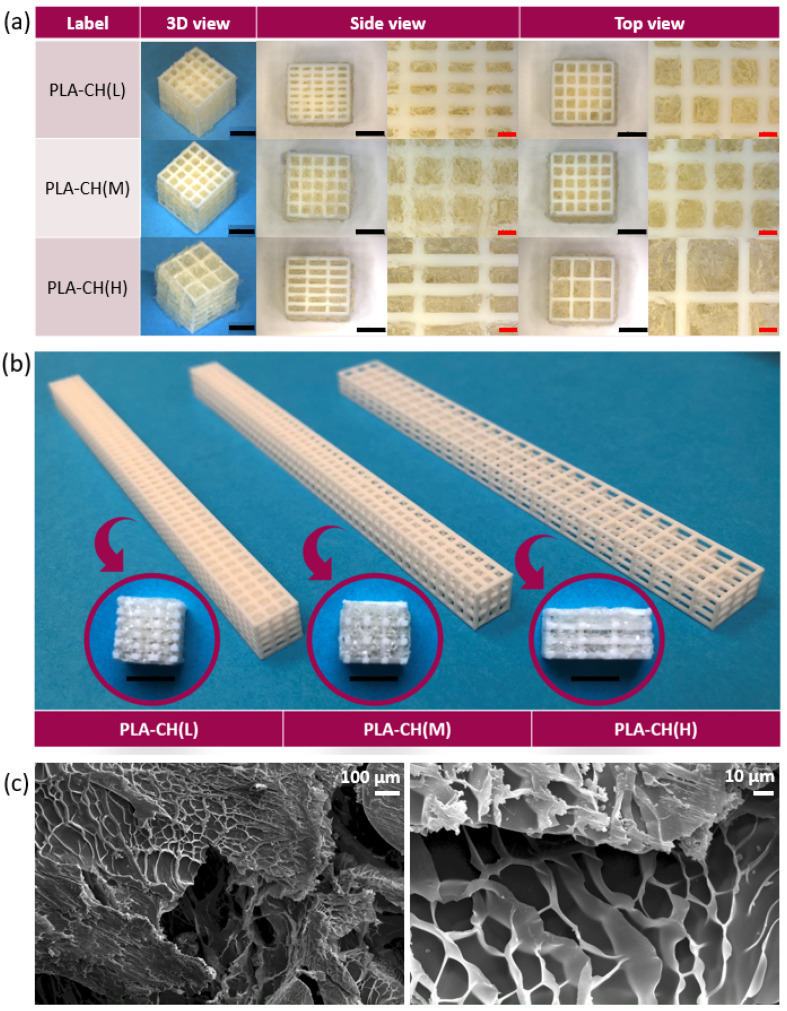
Core–shell scaffolds with low (L), medium (M) or high (H) hydrogel content: (**a**) wet cubic specimens (different viewpoints and magnifications); (**b**) dry slice specimens for in vitro studies, along with the bar-shaped core specimens from which they were obtained. Markers: 5 mm (black) and 1 mm (red). (**c**) Porous structure of the hydrogel observed by SEM.

The appearance of wet core–shell specimens employed for physical–mechanical characterizations is shown in Figure 2a from different viewpoints. Magnified details show that core struts are composed of smaller PLA filaments, with width equal to the nozzle diameter (0.2 mm) in the top view, and height corresponding to the layer thickness (0.1 mm) in the side view. The core struts are surrounded by hydrogel, which is able to penetrate deep into the core through its holes, as further demonstrated by the images of dry sectioned specimens for in vitro studies in Figure 2b; each slice specimen is displayed next to the original core structure of the bar-shaped specimen from which it was obtained. The porous structure of the hydrogel is better shown in images obtained by SEM (Figure 2c), highlighting the presence of a heterogeneous network of interconnected pores, suitable for cell colonization as well as oxygen and nutrients supply.

**Figure 3:**
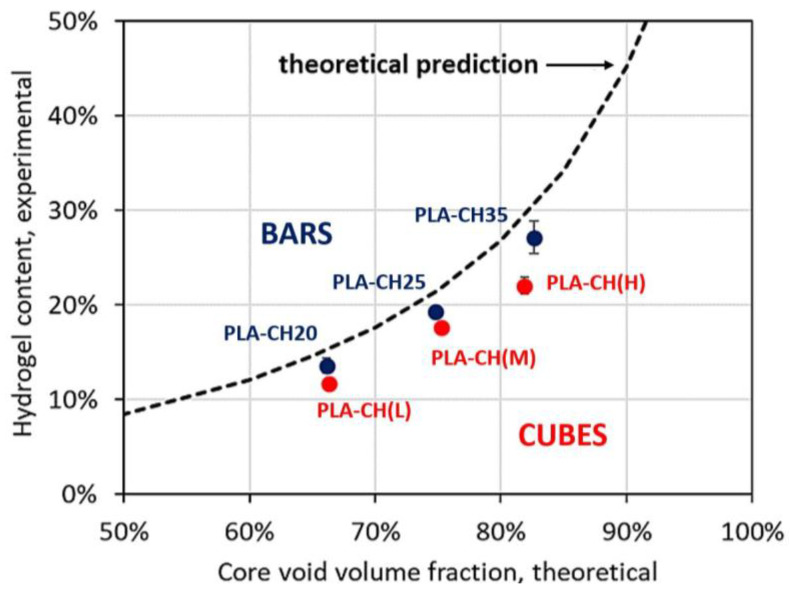
Hydrogel content (weight fraction) versus core void volume fraction for composite scaffolds with either cubic or bar-shaped core; the dashed line represents the theoretical hydrogel content prediction.

Since the shell occupies all the voids in the core, the hydrogel content depends on the void volume fraction in the core, which in turn varies according to the lattice hole dimensions. All these parameters are reported in Table 1 for cubic scaffolds, together with water uptake values after immersion in water for 24 h. The experimental values of the core void fraction are around 70–80% and only slightly higher than those theoretically expected from the CAD models of the lattices. As the core void volume fraction increases, the hydrogel content also grows, leading to correspondingly higher water uptake values, between 100% and 200%. More in detail, the increasing trend of the hydrogel content with the core void volume fraction is shown in Figure 3, for both cubic and bar-shaped specimens. A theoretical trend is also obtained by means of Equation (2), assuming that the porosity of the hydrogel is not affected by the presence of the core struts and that the lattice holes are perfectly filled by the shell. Overall, despite these approximations, the experimental points are only slightly below the theoretical trend (cubes: see Table 1; PLA-CH(L) bars: 13.6 ± 0.6%; PLA-CH(M) bars: 19.2 ± 0.4%; PLA-CH(H) bars: 27.1 ± 1.5%).

**Figure 4:**
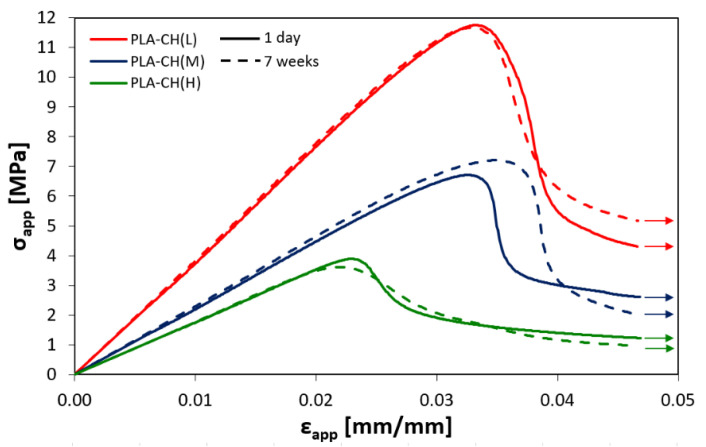
Apparent stress versus apparent strain curves of core–shell scaffolds with low (L), medium (M) or high (H) hydrogel content tested under compression after 1 day or 7 weeks in water at 37 °C.

The mechanical properties of core and core–shell scaffolds were evaluated under compression along the xy direction (more details regarding the effects of the loading direction and of the geometry of the scaffold on its mechanical behavior are reported in a companion paper [37]). Some examples of the obtained stress–strain curves are reported in Figure 4, for PLA-CH(L), PLA-CH(M) and PLA-CH(H) core–shell scaffolds after 1 day in water (continuous lines) and after 7-week hydrolytic degradation experiments reported below (dashed lines). All the curves have the typical characteristics that can be observed in many cellular materials [40]: (i) an initial linear segment up to apparent strain values around 2–5%; (ii) a second region during which the lattice collapses due to buckling phenomena (i.e., unstable bending deformation under compression); (iii) a final part during which the slope rises rapidly because of the structure densification that happens as the walls of the cells come in contact with each other (here not shown). More specifically, the structures are observed to progressively collapse through a series of localized buckling phenomena of individual lattice struts, favored by strut slenderness, and associated with stress peaks in compression curves. The first stress peak is considered as the strength of the specimen, while the apparent modulus of the specimens is evaluated as the slope of the first linear tract of the curves.

**Figure 5:**
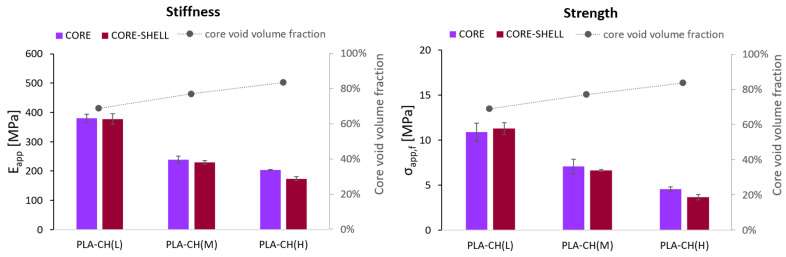
Apparent modulus (E_app_) and apparent failure stress (σ_app,f_) for core and core–shell specimens with low (L), medium (M) or high (H) hydrogel content. The core void volume fraction of the specimens is also reported in both graphs (in gray).

Apparent modulus (E_app_) and apparent failure stress (σ_app,f_) are represented in Figure 5 for PLA-CH(L), PLA-CH(M) and PLA-CH(H) core–shell scaffolds and for the corresponding core structures. Stiffness and strength are found to be similar for core and core–shell scaffolds with the same lattice geometry, suggesting a negligible contribution of the hydrogel shell to the mechanical properties of the composite systems. This may be expected due the low Young’s modulus of the hydrogel, in the order of 10^−2^–10^−1^ MPa [24], which is about 1000 times lower than that of the core. With stiffness values of a few hundred MPa and strength values around 5–10 MPa, the mechanical properties of the hybrid scaffolds are comparable with those of several types of trabecular bone tissue, featuring Young’s modulus values between 10 MPa and 2000 MPa, and compressive strength values between 0.3 MPa and 50 MPa [40]. These results are therefore promising in view of bone tissue engineering applications, in which the scaffolds should temporarily provide sufficient mechanical support while new bone gradually replaces them.

Moreover, the graphs in Figure 5 also show that both the apparent failure stress and the apparent modulus of the specimens decrease as the hydrogel content increases. These trends are the result of the increase in the void volume fraction in the core, indicated through secondary ordinate axes. The core void volume fraction is thus confirmed as an important parameter for tailoring not only the hydrogel content but also the scaffold mechanical properties, according to the specific bone defect to be treated.

### 3.2. Hydrolytic Degradation Experiments

Bone tissue has an excellent self-healing capacity for defects of a few millimeters, but for defects beyond the critical size, a clinical intervention with bone grafts or scaffolds is necessary [6]. In these cases, it is important to prolong the permanence of the scaffold and its mechanical action until a sufficient portion of tissue is regenerated. For this reason, degradation experiments were carried out on core–shell scaffolds immersed in distilled water at 37 °C for 7 weeks, aiming to evaluate their mass loss and the evolution of their mechanical properties over time. Both the rigid core and the bioactive shell need adequate degradation kinetics in order to perform their own function: while the hydrogel should soon start to degrade and be gradually replaced by new bone tissue, the PLA should provide mechanical support for a longer duration.

**Figure 6:**
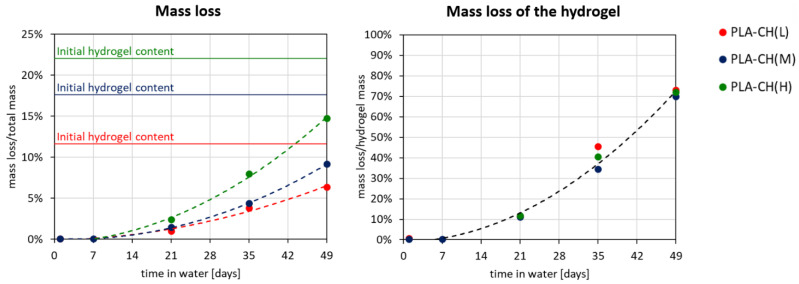
Mass loss versus degradation time for core–shell scaffolds with low (L), medium (M) or high (H) hydrogel content, expressed both as percentage of the initial mass of the dry core–shell specimen and as percentage of the initial mass of the dry hydrogel shell. The trends are fitted with second-order polynomial curves (dashed lines).

The mass loss of the specimens was calculated according to Equation (7) with respect to the initial total mass of the scaffolds, and according to Equation (8) with respect to initial mass of their hydrogel shell; the results are represented in Figure 6 as a function of time and fitted with second-order polynomial lines to better highlight their trends. All the curves show mass loss indicating hydrolytically degradable products, and the degradation rate tends to increase over time. Since PLA hydrolysis at body temperature typically occurs in about 1–2 years [41], its mass loss is expected to be negligible during the first weeks with respect to that of the hydrogel. Indeed, the overall mass loss seems to be proportional to the initial hydrogel content, and the trends of the three scaffold types are observed to overlap when the mass loss is normalized to the initial hydrogel mass (Figure 6b). For all the systems, about 70% of the hydrogel shell is lost after 7 weeks, corresponding to 6%, 9% and 15% of the total mass for PLA-CH(L), PLA-CH(M) and PLA-CH(H), respectively. However, the core–shell structural integrity is maintained for at least 7 weeks and the hydrogel remains grafted throughout the core.

**Figure 7:**
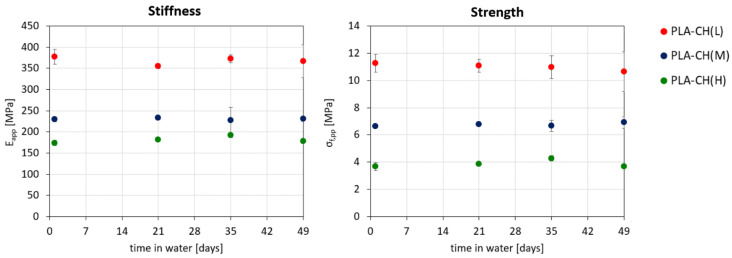
Stiffness (E_app_) and strength (σ_app,f_) as a function of degradation time for core–shell scaffolds with low (L), medium (M) or high (H) hydrogel content.

Despite the negligible PLA mass loss, prolonged immersion in water at 37 °C may still affect the mechanical properties of the lattices. For this reason, the degradation experiments also included compression tests of the specimens, whose apparent failure stress and apparent modulus are reported in Figure 7 as a function of the immersion time; in addition, complete stress–strain curves after 7-week degradation are shown in Figure 4. Both stiffness and strength remain stable up to 7 weeks from the beginning of the tests, when stress–strain curves still display no relevant changes, which is encouraging in view of supporting loads during early bone tissue reconstruction.

As shown previously, scaffold mechanical properties depend on the void volume fraction in the core. Since the void fraction also affects the hydrogel content, a balance should be found between the mechanical properties of the scaffolds and the amount of faster-degrading shell.

### 3.3. In vitro Characterization, Optical Microscopy, and SEM Analysis

**Figure 8:**
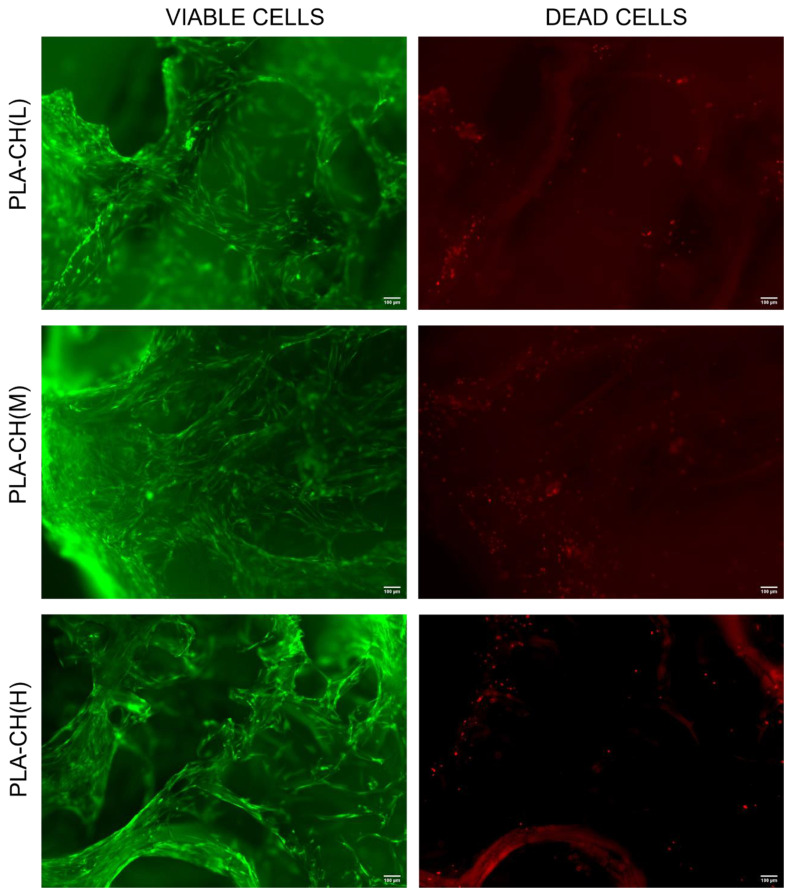
Live/dead staining of BM-hMSCs cultivated in PLA-CH(L), PLA-CH(M), PLA-CH(H) for 28 days in the GM. Scale bar: 100 μm.

After 28 days in GM, cell viability in the scaffolds was evaluated in three replicates. The results achieved from the fluorescence microscopy analysis performed on PLA-CH(L), PLA-CH(M), and PLA-CH(H) highlighted that BM-hMSCs were viable within the scaffolds after 28 days of culture (Figure 8). In more detail, the microscopic data reported confirm homogeneous cell distribution within the scaffolds as well as almost the absence of dead cells (Figure 8).

**Figure 9:**
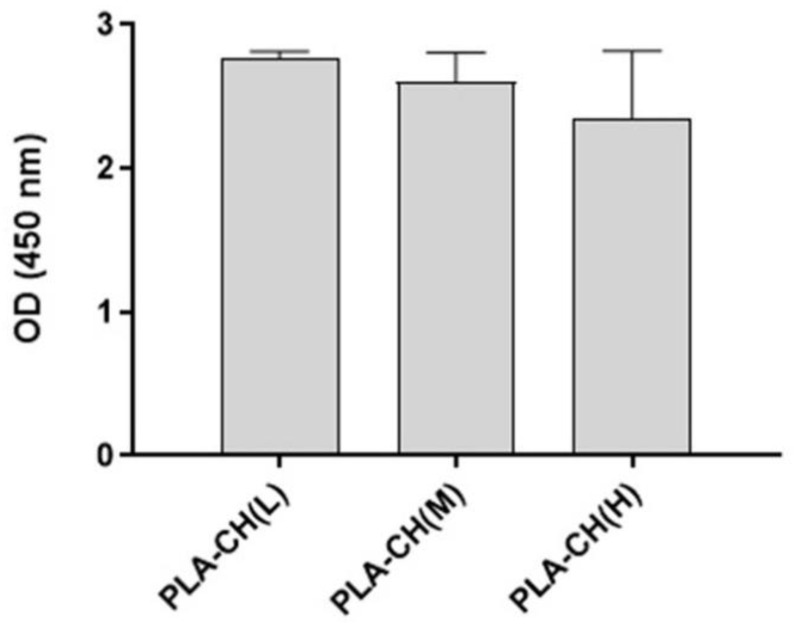
Distribution of absorbance measurements of BM-hMSCs cultivated in PLA-CH(L), PLA-CH(M), PLA-CH(H) in the GM at 28 days measured by the CCK8 assay.

In addition to the data obtained with the live/dead assay, cell proliferation was measured by quantifying viable cell numbers within the scaffolds at 28 days after cell seeding in GM with the Cell Counting Kit-8 colorimetric assay (Figure 9). In more detail, the absorbance average values obtained from the cell-seeded scaffolds with CCK-8 at 28 days were 2.77 ± 0.05 for PLA-CH(L), 2.60 ± 0.20 for PLA-CH(M), 2.35 ± 0.46 for PLA-CH(H). It has been previously shown that the scaffolds alone did not cause any non-specific staining of the scaffolds using the Cell Counting Kit-8 colorimetric assay [39]. Since there were no statistical differences between the scaffolds tested, we considered PLA-CH(L), PLA-CH(M), and PLA-CH(H) comparable, regardless of cells proliferation in the GM. (PLA-CH(L) vs. PLA-CH(M), *p* value: 0.78; PLA-CH(L) vs. PLA-CH(H), *p* value: 0.28; PLA-CH(M) vs. PLA-CH(H), *p* value: 0.58). However, a decreasing trend of cell proliferation, particularly for PLA-CH(H), was revealed after 28 days of culture.

**Figure 10:**
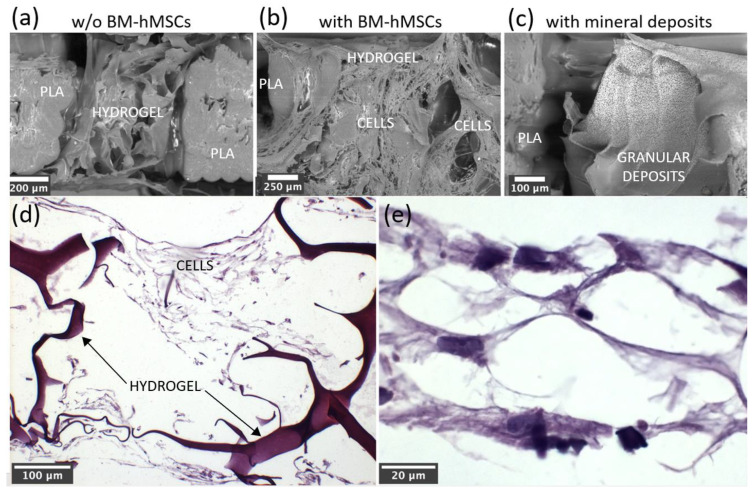
SEM images of PLA-CH(M) without cells (**a**), with BM-hMSCs in the GM (**b**), with BM-hMSCs in the OM and characterized by the production of mineral deposits (**c**), at 28 days and histomorphological analysis at the optical microscope of PLA-CH(M) with BM-hMSCs in the GM at different magnifications (**d**,**e**).

Moreover, SEM analyses were performed on scaffolds without cells (Figure 10a), with BM-hMSCs cultured in GM (Figure 10b,d,e), and with BM-hMSCs cultured in OM (Figure 10c), on day 28 of cell culture. The architecture of the core–shell scaffold is well illustrated in Figure 10a, where the open porous hydrogel is observed to adhere to the struts of the PLA lattice. Figure 10b displays the attachment and spreading of cells in the scaffolds, preferentially occurring inside the hydrogel pores. Cells develop a large spreading area with an elongated fibroblast-like morphology, forming bridges through the pores and showing great affinity with all three types of scaffolds. This is confirmed also by histomorphological analyses at the optical microscope, showing homogeneous colonization of cells inside the hydrogel pores (Figure 10d), as well as their good spreading visible at higher magnifications (Figure 10e). Finally, a preliminary evaluation of the osteogenic differentiation of the BM-hMSCs in PLA-CH(M) was performed. Particularly, the formation of granular deposits was observed using SEM in the scaffolds with BM-hMSCs treated with OM, especially in the hydrogel shell (Figure 10c). According to previous investigations [22,39,42], such deposits could be ascribed to hydroxyapatite formation, as the elemental content measured by X-ray spectroscopy associated with SEM revealed the presence of C, O, N as well as calcium and phosphorous due to mineralization after cell seeding in OM. On the contrary, scaffolds with BM-hMSCs grown in GM, without osteogenic stimuli, didn’t show such a kind of mineral deposition (Figure 10b).

## 4. Conclusions

In this paper, bioresorbable scaffolds with innovative core–shell architecture were realized by grafting a bioactive hydrogel shell onto a stiff 3D-printed PLA core. The two components were selected in order to provide the scaffolds with complementary functions to better match the multiple requirements for bone tissue engineering applications.

The PLA core was designed with lattice structures having different void volume fractions to modulate its stiffness and strength, aiming at providing tailored mechanical support for specific target tissues. These properties resemble those of bone tissue and remain stable in the long term, as demonstrated by hydrolytic degradation experiments.

The hydrogel shell filled the entire void volume of the core lattices, reaching experimental contents in good agreement with the theoretical predictions. The hydrogel interconnected porous structure is preserved in the composite scaffolds and represents the preferred microenvironment for cell colonization and mineralization, as verified during in vitro experiments. Moreover, the hydrogel undergoes gradual degradation, leaving space for new bone tissue formation.

Overall, the core–shell scaffolds proved to be biocompatible and promising for bone tissue regeneration and personalized medicine. In fact, their versatile multi-material design and the use of additive manufacturing allow fine tuning of their physical–mechanical and biological properties. In particular, it is fundamental to reach good balance in mechanical support and regenerative potential by properly designing the core–shell proportions and architecture.

## Figures and Tables

**Table 1:** Dimensions of core holes (w × h), core void volume fraction (theoretical and experimental), hydrogel content (weight fraction) and water uptake of cubic composite scaffolds with low (L), medium (M) or high (H) hydrogel content.

Label	w × h [mm]	Core Void Volume Fraction, th [%]	Core Void Volume Fraction, exp [%]	Hydrogel Content, exp [%]	Water Uptake (24 h) [%]
PLA-CH(L)	1.5 × 0.6	66.3	68.8 ± 0.3	11.6 ± 0.4	102.5 ± 7.4
PLA-CH(M)	1.5 × 1.5	75.3	77.0 ± 0.3	17.6 ± 0.4	137.2 ± 4.3
PLA-CH(H)	3.0 × 1.0	81.9	83.4 ± 0.2	22.0 ± 0.9	201.4 ± 8.9

## Data Availability

Data are contained within the article.
